# Post-Acute Sequelae and Mitochondrial Aberration in SARS-CoV-2 Infection

**DOI:** 10.3390/ijms25169050

**Published:** 2024-08-21

**Authors:** Charles Ward, Beata Schlichtholz

**Affiliations:** Department of Biochemistry, Gdańsk University of Medicine, 80-210 Gdańsk, Poland; charles.ward@gumed.edu.pl

**Keywords:** SARS-CoV-2, PASC, long COVID, mitochondria, mtDNA, autophagy, mitophagy, innate immunity, cell metabolism, reactive oxygen species

## Abstract

This review investigates links between post-acute sequelae of SARS-CoV-2 infection (PASC), post-infection viral persistence, mitochondrial involvement and aberrant innate immune response and cellular metabolism during SARS-CoV-2 infection. Advancement of proteomic and metabolomic studies now allows deeper investigation of alterations to cellular metabolism, autophagic processes and mitochondrial dysfunction caused by SARS-CoV-2 infection, while computational biology and machine learning have advanced methodologies of predicting virus–host gene and protein interactions. Particular focus is given to the interaction between viral genes and proteins with mitochondrial function and that of the innate immune system. Finally, the authors hypothesise that viral persistence may be a function of mitochondrial involvement in the sequestration of viral genetic material. While further work is necessary to understand the mechanisms definitively, a number of studies now point to the resolution of questions regarding the pathogenesis of PASC.

## 1. Introduction

### 1.1. Post-Acute Sequelae of SARS-CoV-2

Referred to variously as “long COVID”, “long COVID syndrome”, “post COVID-19 condition” [1], “post-acute COVID-19 syndrome” [2], or “post-acute sequelae of SARS-CoV-2 (PASC)” [3], PASC is defined as a multi-organ syndrome with a varying yet consistent constellation of symptoms including fatigue, shortness of breath, neurological symptoms of confusion or “brain fog”, stress, anxiety, joint pain, muscle pain, cough, nasal congestion, runny nose, “tightening” chest pain, palpitations, tachycardia and a variety of other symptoms still present 12 weeks since the start of COVID-19 [2,4].

Estimates of PASC prevalence and incidence vary widely between different studies and populations. Conservative estimates suggest that at least 10% of infected people develop PASC [5], and PASC prevalence ranges from 11% to 77.1% of nonhospitalised patients at 60 days post-infection [3,6]. Much research has been undertaken among previously hospitalised patient cohorts with moderate to severe symptoms of acute infection, among whom the prevalence of those suffering sequelae ranges between 32.6% and 87% [7]. A meta-analysis found a global prevalence of PASC of 43% [8]. Geographic variations in PASC prevalence have also been observed, with estimates ranging from 30% in North America to 49% in Asia [8]. Continued research and standardised definitions are vital for assessing this condition. Risk factors for developing PASC include advanced age, female sex, history of smoking, obesity, pre-existing mental health conditions, lack of vaccination against SARS-CoV-2, presence of comorbidities and previous hospitalisation or admission to an intensive care unit [9,10].

### 1.2. Proposed Pathogenesis of PASC

The exact pathogenesis of PASC syndrome has received much discussion, with long-term organ damage [11], immune dysfunction, microbiota disruption, autoimmune interactions, metabolic dysfunction, endothelial dysfunction and post-intensive care syndrome among the foremost proposed theories [9]. The phenomenon of long-term retention of symptoms is not unique to SARS-CoV-19; similar post-acute syndromes have been noted in association with previous coronavirus outbreaks such as severe acute respiratory syndrome (SARS) and Middle East respiratory syndrome (MERS). Post-mortem, histologic patterns of damage in patients dying from acute COVID infection have some similarities to those of patients who die from acute respiratory distress syndrome (ARDS) secondary to H1N1 flu virus, SARS or MERS, having diffuse alveolar damage and perivascular T cell lymphocytic infiltration. In contrast, they also have characteristic endothelial disruption with the presence of intracellular viruses, widespread thrombosis with microangiopathy and sustained angiogenesis at 2.7 times that of patients from control groups [12]. Patients suffered symptoms including abnormalities in pulmonary function [13,14], chronic fatigue [15], significantly increased stress and psychological distress [16] manifesting as increased incidence of psychiatric disease [15], depression [17,18], and overall reduced quality of life. In the aftermath of the more localised SARS and MERS outbreaks, some authors proposed that post-acute sequelae developed as a result of psychological distress [18]. Additionally, pre-existing psychological conditions are linked to the development and exacerbation of psychological symptoms and fatigue after recovering from COVID-19. Individuals who have pre-existing mental disorders like depression and anxiety often have more intense symptoms of depression and anxiety compared with individuals without these conditions [10]. In addition, those with a history of depression were more prone to experiencing symptoms of long COVID that persisted for over 200 days after their initial COVID-19 diagnosis [19]. This implies that existing psychological conditions might play a role in the persistence of long COVID symptoms.

Nalbandian et al. proposed three methods of long COVID syndrome pathogenesis: pathophysiological changes mediated by the specific virus, immunological aberration and inflammatory damage in response to acute infection and expected sequelae of post-critical illness [2]. Venturelli et al. conducted a large-scale study attempting to separate features of long COVID syndrome by method of pathogenesis. Their proposed categorisation grouped symptoms into those explained by post-traumatic stress disorder (including anxiety, flashbacks, and nightmares), those from post-viral chronic fatigue syndrome (such as persistent fatigue, sleep disturbance and headache) and finally those from post-critical illness syndrome (including muscle weakness, respiratory difficulties and cognitive impairment) [20,21]. Surveying 2050 individuals in Tyrol (Austria) and South Tyrol (Italy), Sahanic et al. characterised two distinct phenotypes of acute COVID syndrome, non-specific infection with generalised symptoms and a “multi-organ phenotype” (MOP), suffering from multiple neurological, cardiopulmonary, gastrointestinal and dermatological complaints. They note that patients expressing MOP-type acute infection suffered longer and more damaging post-acute symptoms compared to those with non-specific symptoms [22]. Asthenia, myalgia, cough, dyspnoea, concentration deficit, headache, anosmia, and dysgeusia are the most frequently reported post-COVID-19 symptoms, listed in descending order of prevalence [10]. Both acute and chronic cases of COVID often present with fatigue as a major symptom, with reports showing that up to 46% of patients experience fatigue lasting from weeks to months [23]. Nevertheless, most studies on COVID-19 cohorts have indicated a significant prevalence of ongoing fatigue, with rates varying from 13% to 33% 16–20 weeks after the initial symptoms appeared [23]. It is crucial to acknowledge that numerous severe systemic infections can also lead to fatigue. Moreover, fatigue and cognitive impairment can be the outcome of chronic stress or depression caused by social and economic difficulties, rather than solely being a result of infection [24]. Cognitive dysfunction and neuropsychiatric symptoms are frequently observed alongside persistent fatigue in individuals with COVID-19 [21]. Additionally, fatigue has been associated with decreased physical and mental well-being, underscoring the importance of ongoing monitoring and management of fatigue during the post-COVID-19 era [25]. The screening for post-COVID-19 symptoms, with a specific focus on fatigue, is crucial for early detection, monitoring, and management of long-term effects of the disease.

It was recognised in the early stages of the global pandemic that encephalopathy presented as a rare but significant complication of acute infection [26], and Muccioli et al. propose that similar pathogenesis could be considered responsible for the neuropsychiatric PASC symptoms [27]. While there is little evidence to support the theory that SARS-CoV-2 or previous coronaviruses are direct neuropathogenic, neurological manifestations of the disease, they have nevertheless been features of post-infectious subacute states associated with SA RS, MERS and COVID-19 [28].

### 1.3. Mitochondria: The Cellular Powerhouse at the Crossroads of PASC Pathogenesis

Mitochondrial dysfunction is recognized as a major factor in both acute COVID-19 and the development of PASC. Generating cellular energy heavily relies on the mitochondrial production of ATP. The presence of fatigue, muscle weakness, cognitive impairment, and neurological problems are common symptoms of PASC, which can result from disrupting mitochondrial energy production and the metabolic pathways they are involved in [29]. Mitochondria also play a role in the immune response by generating reactive oxygen species (ROS) that help eliminate pathogens. However, excessive ROS production can result in oxidative stress, which might be a contributing factor to the chronic inflammation observed in PASC. Mitochondria are central to the regulation of inflammation and programmed cell deaths, particularly apoptosis, pyroptosis, and ferroptosis, making them critical players in maintaining cellular homeostasis and responding to cellular stress [30,31,32]. They play a role in amplifying the inflammatory response seen in PASC by increasing the levels of pro-inflammatory cytokines. SARS-CoV-2 infection triggers a complex interplay between the virus and host innate immune response, which is the body’s first line of defence against pathogens or foreign substances [33]. Patients with PASC demonstrate altered fatty acid metabolism, impaired fatty acid oxidation, an accumulation of lactate in the bloodstream during exercise, and increased glycolysis [34,35,36]. The disruption of the NAD + metabolome has been proposed as a pathogenic mechanism in PASC [37]. These findings suggest that the mitochondria were hijacked by SARS-CoV-2 [36]. The importance of mitochondria in PASC cannot be underestimated; recognizing the metabolic abnormalities can pave the way for targeted therapeutic interventions, advancing preventive strategies and enhancing our knowledge of postviral syndromes related to mitochondrial dysfunction.

## 2. Coronavirus Structure, Replication and Persistence

### 2.1. Structure and Replication

Coronaviruses are positive-sense single-stranded RNA (+ssRNA) viruses with a wide degree of variance capable of infecting both humans and a multitude of animal species. Noted for their large >30 kb genomic RNA load, they consist of a number of protein subunits holding varied basic and specialised functions [38]. In total, there are 14 open reading frames (ORFs), 16 non-structural proteins (NSPs) and 9 accessory and structural proteins. The major functions of viral proteins are assisting the virus in binding to and penetrating host cells, participating in the viral genome replication process, and assisting in the assembly and release of new viral particles, while some particles may also interfere with elements of the host immune response [39]. In particular, the structural S “spike” protein forms homotrimers binding the cellular entry receptor and mediates infection, the E “envelope” and M “membrane” proteins form structural assembly components and the N “nucleocapsid” protein encapsulates the viral +ssRNA genetic material [39,40,41].

Initial infection results when the coronavirus S protein binds to cellular entry receptors—most importantly human angiotensin-converting enzyme 2 (ACE2) receptor and cell-surface serine protease TMPRSS2, both found extensively throughout respiratory tract tissues. After cell entry, viral particles have structural proteins removed in endosomes and open reading frames (ORF1a and ORF1b) are immediately translated to NSPs, which will go on to form the perinuclear replication and translation complex (RTC). The RTC produces genetic material within double-membrane vesicles (DMVs) that are translated into more viral particles that will be exocytosed from the infected cell [38]. Cryo-electron microscopy indicates that perinuclear DMVs have a hexameric NSP3-constituted molecular pore, which allows replicated viral genetic material to be exported for translation and packaging of new virions [42].

### 2.2. Persistence and Multi-Organ Distribution of SARS-CoV-2 RNA and Proteins

SARS-CoV-2 RNA and replicating viruses are not fully cleared after acute infection, indicating that reservoirs of viral material persist long after infection [43]. However, it is important to note that the interpretation of viral persistence data requires caution. The great persistence of this viral RNA is surprising because endogenous mRNA is typically degraded in an efficient manner by nuclease enzymes, with various studies estimating the median half-life for all genes between 7 [44] and 10 h [45]. Yang et al. showed that alternative transcripts have higher degradation rates owing to specific sequences within retained introns or alternatively spliced exons [45]. Sharova et al. demonstrated that genes involved in the regulation of the cell cycle, transcription, and apoptosis had the shortest half-lives while genes regulating cellular metabolism, protein biosynthesis and maintenance of the cytoskeleton and extracellular matrix were the most persistent [44].

Multiple studies have confirmed the presence of +ssRNA, the full S protein, spike S1 subunit and N protein long after acute symptoms have resolved [46,47]. Craddock et al. demonstrated the persistence of both viral RNA and the S protein in both convalescent patients and patients with long-term sequelae, the latter demonstrating significantly higher levels of both RNA and S protein than convalescent patients returned to health [47]. Multi-organ involvement seems highly likely with regard to both acute and post-acute symptoms, especially in light of viral RNA and spike proteins being localised throughout the blood serum [46], gastrointestinal tract [48], gallbladder [49], tonsils [50], and central nervous system [51]. While these findings suggest multi-organ involvement, it is crucial to distinguish between localized persistence such as in the nasal cavity and systemic effects or neurovascular invasion. It is worth noting that PCR positivity, especially from nasal swabs, may not accurately reflect ongoing active infection or systemic effects as it can detect viral RNA long after the virus is no longer replication-competent [52,53]. While some studies provide evidence for neurovascular invasion and varial mediation of multi-systemic symptoms in acute infection, the presence of a persistent infection in the nasal cavity in patients with mild or asymptomatic disease does not imply neurovascular invasion and systemic effects [54].

A number of early pandemic autopsies in 2020 localised viral RNA and protein within the central nervous system of patients succumbing to acute infection [55], while Stein et al. localised viral RNA and protein to a number of locations, most significantly the cervical spinal cord, cerebellum, basal ganglia and hypothalamus [51], and others have determined the presence of viral genetic material in cerebrospinal fluid via PCR [56]. Autopsies undertaken in 2020 revealed that the presence of viral components accompanied profound histologic change including perivascular leukocytic infiltration, hypoxic change, focal haemorrhage, necrosis and oedema [12]. This correlates with a large-scale longitudinal study conducted by the UK Biobank where two sessions of magnetic resonance imaging were conducted on 401 individuals who tested positive for SARS-CoV-2 after their first imaging, with the results compared with 384 control individuals who did not test positive. Overall, individuals contracting SARS-CoV-2 infection showed pathological change consistent with tissue damage to the primary olfactory cortex, a greater degree of cortical atrophy and a greater-than-expected loss of cognitive function than would be expected in this cohort [57].

While these studies provide ample evidence for neurovascular invasion and viral mediation of multi-systemic symptoms in acute infection, nothing provides better evidence for the persistence of neurological post-acute sequelae than the long-term retention of viral RNA and spike proteins in highly variable tissues around the body. Roden et al. demonstrated that post-mortem lung tissue retained viral RNA up to 174 days after acute infection [58], while Zollner et al. demonstrated viral RNA and nucleocapsid protein within the gut mucosa of 32 patients with irritable bowel syndrome averaging 219 days since acute infection diagnosis [59]. Viral RNA has been further localised in the skin and appendix of a patient 426 days after symptom onset [60].

Clearly, viral genetic material persists many orders of magnitude longer than endogenous mRNA. The long latent period suggests that patients with post-acute sequelae harbour actively replicating viral reservoirs; however, the highly varying quantities of genetic material present indicates that the size or activity of these reservoirs is both variable among the patient cohort and can be expressed irregularly at different times of post-acute activity [43]. In conclusion, while evidence suggests that SARS-CoV-2 can persist in various body sites beyond the acute phase, the clinical significance of these findings and their relationship to PASC requires further investigation.

## 3. Autophagy, Mitochondrial Damage and Innate Immune Response

### 3.1. Autophagy and Mitophagy

The relationship between autophagy and PASC is currently a focus of ongoing research, with several hypotheses suggesting how impaired autophagy could play a role in the persistent symptoms associated with this condition. Autophagy (or autophagocytosis) is a highly conserved cellular adaptation process seen in ageing, neurodegeneration, cancer and infection [61,62]. This natural process of cell degradation eliminates unnecessary or dysfunctional components using a regulated mechanism that depends on lysosomes. Unstressed cells typically have minimal autophagy, yet it is rapidly enhanced in cases of nutrient deprivation, infection, and cellular damage. Initially, autophagy is characterised by the formation of a double-membrane phagophore (PG) vesicle within the cytosol allowing cellular structures to be identified for disposal. PG vesicles mature to fuse with lysosomes resulting in degradation and recycling of cellular structures [63]. Autophagy is essential in balancing innate immune inflammatory responses. The protective effect is typically exerted through the regulation of inflammatory responses and maintenance of cellular homeostasis during stress. By delivering viruses and viral proteins to lysosomes for degradation, autophagy acts as a defence mechanism against viral infection in cells [64]. Although autophagy is primarily recognized for its role in protecting host cells from viruses, it can also play a dual role by either defending against viruses or aiding in viral replication during acute infections [65,66]. SARS-CoV-2 induces incomplete autophagy, promoting autophagosome formation but inhibiting autophagosome-lysosome fusion, which facilitates its replication [67,68]. This process is mediated by viral proteins, particularly ORF3a, which interacts with autophagy regulators [68]. Conversely, findings from other studies indicate that SARS-CoV-2 disrupts the autophagic flux as a means to evade destruction and promote exocytosis, ultimately facilitating the release of the virus [69]. The virus interferes with multiple metabolic pathways, including polyamine metabolism, and protein translation to limit autophagy [70]. The dysregulation of autophagy was associated with excessive inflammation, which was observed in severe cases of COVID-19 and could potentially contribute to the persistent inflammatory state seen in PASC.

**Figure 1:**
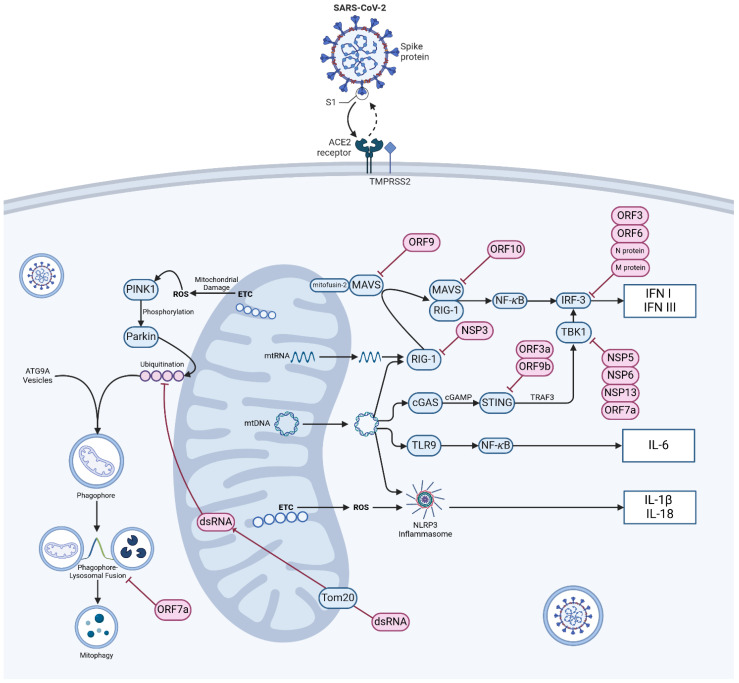
Mechanism of SARS-CoV-2 entry, mitochondrial damage sensing and innate immune dysregulation. Viral particles enter cells via the angiotensin-converting enzyme 2 (ACE2) receptor and TMPRSS2 protease. In normally functioning cells various mechanisms exist to warn of mitochondrial damage and viral infection, most notably via retinoic acid inducible gene-1 and mitochondrial antivirus signalling protein (RIG-1-MAVS), cyclic GMP–AMP synthase and stimulator of interferon genes (cGAS-STING) signalling, Toll-like receptor-9 (TLR9) and activation of the NOD-, LRR -and pyrin domain containing protein 3 (NLRP3) inflammasome. Damage to mitochondria resulting in the production of ROS initiates mitophagy via the PTEN-induced kinase 1 (PINK1)-Parkin E3 ubiquitin ligase pathway. Infection of cells by SARS-CoV-2 disrupts these axes at a number of control points and causes widespread aberration to systems of innate immunity. Abbreviations: ACE2—angiotensin-converting enzyme 2; ATG9A—autophagy-related protein 9A; cGAMP—cyclic GMP-AMP; cGAS—cyclic GMP-AMP synthase; dsRNA—double-stranded RNA; ETC—electron transport chain; IL-1β—interleukin 1 beta; IL18—interleukin 18; IL-6—interleukin 6; IFN I—type I interferon; IFN III—type III interferon; MAVS—mitochondrial antiviral-signalling protein; NFκB—nuclear factor kappa-light-chain-enhancer of activated B cells; NSP—non-structural protein, ORF—open reading frame; Parkin—E3 ubiquitin-protein ligase parkin; ROS—reactive oxygen species; STING—stimulator of interferon genes; TBK1—TANK-biding kinase 1; TMPRSS2—transmembrane protease, Serine 2; TLR9—Toll-like receptor 9; Tom20—translocase of outer mitochondrial membrane 20; TRAF3—TNF receptor-associated factor 3; mtDNA—mitochondrial DNA. Pink objects indicate material of viral origin, blue objects indicate self-material. (Created with BioRender.com).

Mitophagy is a specific form of autophagy where damaged mitochondria are degraded within autophagosomes and act as a form of “quality control” over damaged mitochondria that inappropriately produce ROS or leak internal contents [63]. It is controlled within the cell by the PINK1-Parkin pathway and the accumulation of mitochondrial p62 also known as sequestosome-1 [71]. PTEN-induced kinase 1 (PINK1) is a serine/threonine kinase that phosphorylates both ubiquitin and Parkin (an E3 ubiquitin ligase). Active Parkin attaches serial ubiquitin chains to mitochondrial outer membrane proteins marking the defective organelle for destruction (Figure 1) [72,73]. The damaged organelle is then packaged into ATG9a integrated vesicles, upon which the “phagophore” can then be fused with lysosomes and degraded [74]. p62 is an autophagy receptor protein that aggregates around damaged or misfolded proteins, facilitating their degradation through autophagy. Defective p62 activation is associated with proteinopathies such as Huntingdon’s disease [75] while the PINK1-Parkin pathway of mitochondrial degradation relies on the recruitment and activation of p62 [76]. p62 links ubiquitinated proteins to the autophagy machinery by interacting with the microtubule-associated protein light chain (LC3) and helps in the formation of autophagosomes [77]. By ensuring the quality control of mitochondria and eliminating viral dsRNA, mitophagy plays a key role in defending against SARS-CoV-2 through the activation of the PINK1-Parkin pathway [78]. It has also been shown that SARS-CoV-2 infection disrupts the interaction between p62 and LC3 leading to the inhibition of mitophagy. Thus, SARS-CoV-2 infection impairs mitophagy completion, potentially as an anti-autophagy strategy [78]. Impaired mitophagy leads to the accumulation of damaged mitochondria, resulting in increased ROS production, chronic inflammation and organ failure.

### 3.2. Mitochondrial Fusion and Fission

It is common practice to represent mitochondria as static organelles enclosed within the cytoplasm of a cell, isolated from each other and individual in form and function. Under-represented in the literature is an understanding of the highly dynamic nature of the cellular mitochondrial mass. Individual mitochondria exist within a continuum of fusion and fission to form new organelles and split those that have become too large. This cycle functions to produce a well-controlled mass of mitochondrial material of regulated size and efficiency catering to the specific energy requirements of the cell, which, when surplus to requirements or no longer functional, can be catabolised to its component parts.

Fusion is mediated by highly conserved outer membrane GTPases mitofusin-1 and -2 and inner membrane protein optic atrophy 1 (OPA1). The role of OPA1 is to uphold the structure of the membrane and provide protection for mtDNA. Mitochondrial fragmentation occurs when any of these proteins are removed, thus allowing the clearance of damaged mitochondria via mitophagy [79,80]. Although the specific molecular triggers for OPA1 processing are not well understood, it is clear that both apoptosis activation and disruption of mitochondrial membrane potential result in the cleavage of OPA1 [81,82]. During fusion, there is a rapid exchange of components like metabolites and soluble proteins, whereas membrane-embedded proteins and mtDNA spread at a slower rate. The fusion of mitochondria helps minimize heterogeneity of the content, making it the first line of defence against dysfunction [83,84].

Fission is mediated by the outer membrane protein mammalian homologue of yeast FIS1 (hFIS1) and dynamin-related protein 1 (DRP1) in the cytosol [85]. The process is well regulated as cells with a low energy balance undergo changes allowing mitochondria to become more granular, separate from each other and gain higher surface area [86]. When visualised using fluorescent biomarkers localised within mitochondria the process is seen to be highly dynamic, providing a balance between the rate of fusion and fission allowing self-regulation of mitochondrial length and motility. The probability that a single mitochondrion will undergo fission is largely a product of length, while the probability that it will undergo fusion is largely a product of motility [87]. Imbalance in the fusion–fission cycle can lead to the pathological generation of excessively large “megamitochondria” [86], the formation of which has been associated with various cardiac, renal, hepatic and neurodegenerative diseases [88].

Cellular mitochondria have a dynamic nature governed by repeated, cyclical fusion–fission episodes, which must remain in balance for the proper function of the cell to continue. We can, therefore, dispel the orthodox view that singular organelles are largely separate in function and efficiency and, once no longer functional, are degraded by mitophagy. Instead, pathological disturbances resulting in damage to mitochondrial components have the potential to affect the whole mitochondrial compartment, while individual components of the mitochondrial compartment are degraded and recycled as necessary. Mitochondrial fusion–fission processes have been found to be disrupted in several diseases, including neurodegeneration, obesity, type II diabetes, and COVID-19 [89,90]. Disruption in mitochondrial dynamics can cause oxidative stress, mitochondrial disfunction and dysregulation of the innate immune response during infection by SARS-CoV-2. During the early stages of infection, cells exhibit modifications in mitochondria characterized by thinner and elongated structures, suggesting morphological and dynamic fusion–fission related change [91]. The virus induces a rise in mitochondrial transmembrane potential resulting in elongated mitochondria and increased ATP synthesis, potentially contributing to viral replication and disease progression [92]. The virus therefore exacerbates the severity of the disease by promoting the fusion and elongation of mitochondria, more effectively replicating within the host cells [90]. SARS-CoV-2 changes mitochondrial dynamics, affecting fission, fusion and membrane potential. Therefore, it affects the body’s energy production, metabolism, and innate immune system signalling [93].

### 3.3. Mitochondrial Control of Inflammation

Inflammation is a complex biological response to infection or tissue damage, involving the coordinated action of various mediators to defend pathogens, repair damaged tissues, and prevent further injury [94]. Mitochondria can act as signalling platforms for controlling inflammation through various mechanisms. The dysfunctional mitochondria can lead to oxidative stress, which subsequently triggers inflammation and tissue remodelling [95]. The release of mitochondrial constituents and metabolic products can act as damage-associated molecular patterns (DAMPs). Examples of DAMPs include N-formyl peptidase, mtDNA, the DNA derived from viral infections or single-stranded viral RNA [96]. Fragmented or oxidized mtDNA can trigger inflammation via the activation of three main pro-inflammatory mechanisms: (1) Toll-like receptors (TLR9 signalling pathway), and (2) cytosolic cyclic GMP/AMP synthase—stimulator of interferon genes DNA-sensing system (cGAS-STING pathway), and (3) nucleotide-binding oligomerization domain-like receptor family pyrin domain containing 3 (NLRP3) inflammasome (NLRP3-mediated inflammation) (Figure 1).

Extracellularly, mtDNA activates inflammatory responses via neutrophil-bound pattern recognition receptor (PRR) Toll-like receptor 9 (TLR-9) [97], and intracellular, endosomal TLR-9 is an element of the important signalling pathway that recognizes unmethylated CpG motifs in DNA commonly found in bacteria, viruses [98], and also mtDNA [99]. Activation of TLR9 by mtDNA recruits an adaptor protein MYD88, which then initiates a signalling pathway resulting in the activation of nuclear factor κB (NF-κB) and triggering inflammatory and antiviral responses. Intracellularly, mtDNA release into the cytosol is sensed by the DNA sensor cGAS and triggers the cGAS-STING pathway, leading to the expression of type I interferon and inflammatory cytokines like TNF and interleukins [100]. STING recognises the presence of viral genetic material and, via a series of second messengers including TANK-binding kinase 1 (TBK1), causes phosphorylation of IRF3 to drive the transcription of type I IFNs such as IFNα and IFNβ and starts the process of autophagy (Figure 1) [101]. Not only IRF3 but also the cGAS-STING pathway can activate NF-κB through alternative mechanisms [102]. There is significant evidence that suggests mtDNA acts as an endogenous activator of inflammasomes. The inflammasome, consisting of cytoplasmic multiprotein complexes, is a critical component of the innate immune system’s protection against pathogens. “Canonical” inflammasomes are capable of activating caspase-1 and are activated either by infection, endogenous proteins associated with mitochondrial damage caused by reactive oxygen species (ROS), other damage-associated molecular patterns (DAMPs) or pathogen-associated molecular patterns (PAMPs) [103,104]. Several types of inflammasomes have been discovered, such as NLRP1, NLRP3, NLRC4, and AIM2 [105]. The clevage of pro-caspase 1 into caspase 1 triggers the activation of inflammasomes and the release of pro-inflammatory cytokines.

There is a possibility that the process of autophagy is influenced by mtDNA, which can lead to the overproduction of inflammatory mediators and activation of apoptotic signal pathways, and the interaction between autophagy and apoptosis further supports this connection [106]. Defective autophagy (mitophagy) allows the accumulation of damaged mitochondria and the release of mtDNA, triggering inflammatory pathways like cGAS-STING and NLRP3 [107,108]. The cGAS-STING pathway activated by cytosolic mtDNA can induce apoptosis through various mechanisms including ER stress and NF-κB activation [109]. Alternatively, autophagy could act as a negative regulator of mtDNA-induced inflammatory responses by inhibiting TLR9 overexpression during inflammation [110].

**Figure 2:**
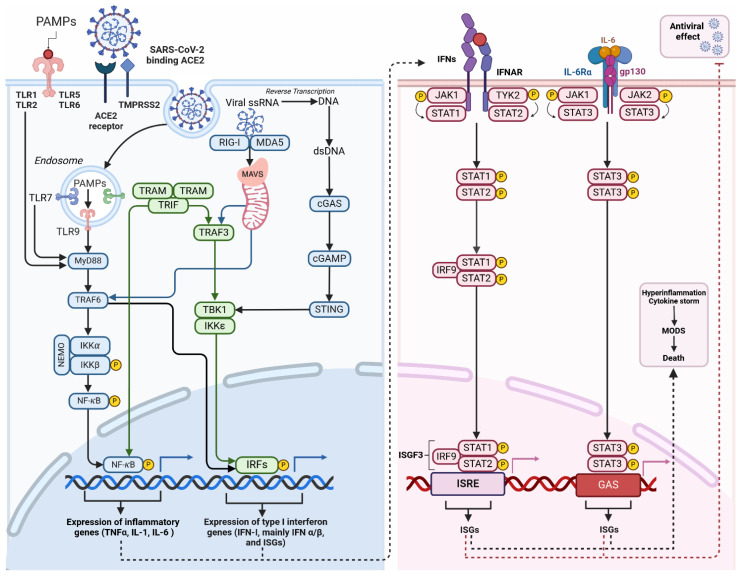
Inflammation triggered by macrophage after SARS-CoV-2 infection. Activation of Toll-like receptors (TLRs) occurs when they recognise pathogen-associated molecular patterns (PAMPs). These patterns are typically found in foreign organisms, such as bacteria and viruses. TLR localisation can occur on either the cell surface (TLR-1, -2, -4, -5, -6, -10) or in intracellular compartments such as endosomes (TLR-3, -7, -8, -9). The ability to recognise viral single-stranded RNA (ssRNA) implies its potential for SARS-CoV-2 clearance of SARS-CoV-2. These receptors detect signals and initiate NF-κB and IRF activation. Activation can occur through the MyD88-dependent and MyD88-independent pathways, ultimately leading to the expression of cytokines and interferon (IFN-I). The binding of viral RNA to RIG-I or MDA5 prompts the creation of MAVS polymers in mitochondria, followed by the subsequent attachment of TRAFs. TRAFs activate the NF-κB, and interferon regulatory factors (IRFs), mainly IRF-3 and IRF-7. This process leads to the expression of antiviral interferon-stimulated genes (ISGs) and pro-inflammatory cytokines. Cytosolic DNA sensors, primarily cGAS detect viral DNA, including dsDNA from DNA viruses or reverse-transcribed DNA from retroviruses. Upon binding to dsDNA, cGAS becomes activated and catalyses the production of a second messenger cyclic GMP-AMP (cGAMP). cGAMP binds and activates STING, which recruits and activates TBK1. Activated TBK1 phosphorylates the transcription factor IRF3 leading to the expression of ISGs. Activation of the JAK-STAT pathway by IFN and cytokines initiates the innate immune response against viral infections. The role of this pathway is critical in various physiological and pathological processes like cancer development, inflammation, tissue damage, and viral infections. The binding of Type I IFN to IFNα receptors (IFNAR) activates JAKs, which in turn phosphorylates and moves STATs to the nucleus. This process leads to the expression of antiviral ISGs. Once the STAT1-STAT2 heterodimer is formed, it binds to IRF9 to create the transcriptionally active ISGF3. The early immune response to viral infections heavily relies on the activation of the Jak2/STAT3 signalling pathway by Il-6, which facilitates virus clearance through neutrophils. The IL-6 protein binds to the IL-6R receptor, which is composed of the IL-6α receptor molecule and the gp130 signal transducer, allowing cell signalling. JAK2 activation induced by IL-6 through gp130 results in the activation of STATs, particularly STAT3. Abbreviations: ISGs—interferon-stimulated genes; cGAMP—cyclic GMP-AMP; cGAS—(cyclic GMP-AMP synthase); GAS—interferon-activated site; IFNs—interferons; IKKα—IκB kinase α; IRF9—interferon regulatory factor 9; ISGF3—INF-stimulated gene factor 3; ISGs—interferon-stimulated genes; ISRE—interferon-stimulated response element; JAK—Janus kinase; MDA5—melanoma differentiation-associated protein 5; MODS—multiple organ dysfunction syndrome; MyD88—myeloid differentiation primary response 88; NEMO—NF-κB essential modulator; PAMPs—pathogen-associated molecular patterns; RIG-I—retinoic acid-inducible gene I; STAT—signal transducer and activator of transcription; STING—stimulator of interferon genes; TBK1—TANK-binding kinase; TRAF—tumour necrosis factor receptor-related factor; TRAF6—tumour necrosis factor receptor-related factor 6; TRAM—trif-related adaptor molecule; TRIF—domain-containing adaptor protein inducing interferon β; Tyk2—tyrosine kinase 2. Different colours in the figure represent various components of the signaling pathways–blueindicates MyD88-dependent and cGAS-STING pathways, green represents IRF, and pink represents the JAK/STAT pathway. c. (Created with BioRender.com).

MAVS, also known as a mitochondrial antiviral signalling protein, is a key component in the innate immune system’s response to viral infections [111]. Localised in the outer mitochondrial membrane, MAVS plays a role in activating type I interferon production during viral infections, acting downstream of the cytosolic RNA sensor RIG-I (Figure 2). Currently, RIG-I and MDA-5 are identified as members of RIG-I-like receptor family (RLR) [112]. In the resting state, MAVS is bound to mitofusin-2 and located in the mitochondrial membrane. Activated RIG-1 and associated chaperone proteins form a complex with MAVS, which, dissociating from mitofusin-2 and the outer mitochondrial membrane, is free to translocate and activate the transcription of antiviral proteins [113]. RIG-I-MAVS (for cytosolic RNA sensing) and cGAS-STING (for cytosolic DNA sensing) pathways are regulated by a complex series of ubiquitination and de-ubiquitination reactions [114] and it is notable that both converge on interferon regulatory factor 3 (IRF-3) [101]. The activation of these pathways triggers the synthesis of interferon and pro-inflammatory cytokines. These molecules are crucial for establishing an antiviral state in infected cells and neighbouring cells [115]. Furthermore, they are responsible for recruiting and activating other components of the immune response. The immune response is tightly regulated to prevent excessive inflammation and tissue damage.

SARS-CoV-2 infection triggers a robust inflammatory response particularly in interstitial macrophages (IMs), contributing to hyper-inflammation in COVID-19 patients [116]. Moreover, macrophage dysregulation plays a crucial role in COVID severity and the pathogenesis of PASC [117]. The release of pro-inflammatory cytokines and chemokines contributes to the “cytokine storm” associated with severe COVID-19 [118]. Elevated levels of pro-inflammatory cytokines, including IL-1β, IL-6, TNF-α, have been associated with PASC symptoms and disease severity [119,120]. The IMs’ response has the potential to impair lung function and spread inflammation to other organs [116]. Prolonged immune activation can result in neuroinflammation, immunothrombosis, and dysfunction of multiple organs [121,122]. The massive activation of macrophages is a major factor in the development of systemic complications including acute respiratory distress syndrome (ARDS) and idiopathic pulmonary fibrosis (IPF) associated with SARS-CoV-2 [118,123].

### 3.4. Viral Interference in Innate Immune Response

The SARS-CoV-2 virus employs a range of strategies to disrupt and avoid the innate immune response of the host, with a particular focus on the interferon (IFN) response. Proteins immediately translated from viral open reading frames interfere at multiple levels of anti-viral interferon responses [124]. It has now been recognised that SARS-CoV-2 and a variety of other coronaviruses are capable of de-ubiquitination and can produce dysregulation at multiple layers of interferon-mediated innate immune response. In SARS-CoV-2, this is accomplished by the papain-like protease (PLpro) domain of NSP3, which acts as a deubiquitinating enzyme and is capable of a wide pattern of disruption to interferon production and response [125]. Indeed, Zhao et al. went as far as to characterise the entire interplay between SARS-CoV-2 and infected hosts as a battle for dominance over E3 ubiquitin ligase (one of a triad of ubiquitinating enzymes) and de-ubiquitinating enzymes [126]. Through deubiquitinating the proteins involved in IFN signalling pathways, PLpro has the ability to inhibit the synthesis of IFN-β and suppress the downstream antiviral effects of IFN-stimulated genes (ISGs). Inhibition of type I interferon signalling, can lead to increased viral load and persistent symptoms contributing to severe COVID-19 and potential long-term effects [127,128].

A surprisingly large selection of viral proteins have secondary roles interfering with innate cellular antiviral processes. Immediately on cellular access NSP1 decreases cytoplasmic translation of type I (including IFN-α and IFN-β) and III interferons and favours cellular translation of viral mRNA over cellular mRNA [28], ORF-3 [129], ORF-6 and N protein all inhibit phosphorylation of interferon regulatory factor-3 (IRF-3), preventing its translocation from cytoplasm to nucleus where it should act as a transcription factor activating interferon production [124]. Similarly, the SARS-CoV-2 M protein inhibits the production of IFN-β and other type I interferons by inhibiting IRF3 phosphorylation and nuclear translocation [40]. The N protein, meanwhile, has also been found to dramatically inhibit cellular response to interferon proteins. Cells with the viral N protein have reduced expression of DNA binding nuclear factor protein NF-κB, here responsible for activating the expression of interferon anti-viral genes via interferon-stimulated response element (ISRE) [124]. Viral elements interfere with the cGAS-STING-TBK1 axis of IRF-3 activation at numerous levels, with ORF3a and ORF9b interfering with STING activation and ORF7a, NSP5, NSP6 and NSP13 interfering with TBK1 activation [101]. In a 2020 study, Jiang et al. determined that viral accessory protein ORF9b interfered with MAVS in the outer mitochondrial membrane, also inhibiting type I IFN production [130].

Viral infections can change the shape and function of mitochondria and these changes can lead to cell death and affect how cells produce energy and defend against viruses. Mitochondria have a vital role in defending the body against viruses and understanding how viruses and mitochondria interact could assist in finding new ways to treat disease. Studies demonstrate that viral infection interferes with the anti-inflammatory effects of IL-6, a cytokine that, via the JAK/STAT pathway, activates genes involved in differentiation, survival, apoptosis and proliferation [131] and viral targeting of parts of the JAK/STAT pathway results in both interferon dysfunction and insensitivity to IL-6 [132]. This, accompanied by viral-mediated mitochondrial dysfunction leading to activation of the TLR-9-NFκB-IL-6 axis, potentially explains why severe acute COVID-19 infection is characterised by high circulating IL-6, sustained cytokine production and hyper-inflammation [133]. Evidently, the SARS-CoV family has evolved to evade interferon-mediated cell death by both inhibiting the production of IFNs and inhibiting cellular response to their activation at multiple levels.

## 4. Alteration to Cellular Metabolism in SARS-CoV-2 Infection

### 4.1. Functional Change in Energy Production in Infected Cells

During SARS-CoV-2 infection, infected cells can undergo significant functional changes, including alterations in energy production. These changes are part of host cell responses to the virus’s strategies to exploit cellular machinery for its own replication. Recent research has uncovered notable changes in metabolism linked to energy deficiency in patients with PASC. Persistent fatigue, a hallmark of PASC, is directly linked to impaired generation of mitochondrial ATP [134]. In another study, patients showed a decline in their exercise capacity, primarily due to weakened skeletal muscle function and mitochondrial dysfunction [135]. SARS-CoV-2 can cause mitochondrial dysfunction, impacting multiple metabolic pathways such as glucose and fatty acid metabolism, as well as amino acid turnover. This ultimately leads to a decrease in ATP production and a lower metabolic rate. In addition, the virus’s ability to suppress the expression of genes that play a crucial role in oxidative phosphorylation (OXPHOS) causes a reduction in energy production [136].

Recent studies have highlighted significant changes in the glycolytic pathway associated with COVID-19 and PASC. SARS-CoV-2 induces a metabolic change that resembles the Warburg effect seen in cancer cells, favouring aerobic glycolysis [137]. This metabolic reprogramming leads to increased glucose uptake, elevated lactate production and reduced ATP production [138,139]. Similar to the neoplastic Warburg effect, it has been hypothesised that “Warburg-like” reconfiguration of cellular glucose metabolism enables virus-invaded cells to produce more biosynthetic substrates providing greater capacity for virion manufacture, viral replication in host cells likely being supported by alteration of cellular glucose metabolism [140]. A metabolic shift in immune cells and lung epithelial cells caused by SARS-CoV-2 infection, which results in higher levels of glycolysis (hyperglycolysis), leads to the excessive production of pro-inflammatory cytokines and ROS [137]. Complementary treatments for severe COVID-19 patients such as drugs like 2-deoxy-D-glucose, GLUT1 inhibitors, high-dose vitamin C, and high-dose N-acetylcysteine, have the potential to interfere with changes in the glycolytic pathway and offer clinical benefits [137].

Codo et al. isolated and sequenced RNA from bronchoalveolar lavage (BAL) of patients with severe infection and determined that circulating monocytes reconfigured their energy-producing metabolism to overwhelmingly glycolytic means while simultaneously upregulating expression of ACE2 receptors [139]. Similar levels of mitochondrial derangement were found by Ajaz et al. in 2021 [36]. These early studies support observations regarding the interesting changes in the metabolic profile of classically and alternatively activated macrophages. Classically activated (M1) pro-inflammatory macrophages rely on aerobic glycolysis to provide rapid and efficient responses to microbes, whereas alternatively activated (M2) anti-inflammatory macrophages have a metabolic profile based on oxidative phosphorylation [141]. Activation is determined by two mutually inhibitory arginine-requiring pathways: M1 macrophages via the inducible nitric oxide (NO) synthase pathway involving PI3K/AKT/mTOR and M2 via arginase and AMPK signalling [142]. In M1-activated macrophages, the presence of NO inhibits mitochondrial aconitase (ACO2) and inactivates isocitrate dehydrogenase 2 (IDH2) resulting in a curtailing of the TCA cycle, exfiltration of citrate from the mitochondria and stabilisation of hypoxia-inducible factor-1α (HIF-1α) [140]. Interestingly, the pro-inflammatory immune profile reported in the metabolomic study in PASC patients with chronic fatigue syndrome suggests an activation of M1-type macrophages in those patients [134].

Metabolomic studies revealed significant alterations in PASC patients including mitochondrial dysfunction, redox imbalance, and impaired energy metabolism [143]. The study revealed metabolic abnormalities, with notable changes found in amino acids, acylcarnitines, and lipids [134,143]. In the study by Saito et al., metabolomic analysis of plasma samples using chemical isotope labelling liquid chromatography-mass spectrometry (CIL LC-MS) showed various metabolic alterations in long COVID patients compared to the recovered and healthy control group, suggesting a dysregulated immune response and impaired mitochondrial bioenergetics in long COVID [134]. Surprisingly, there were no significant differences in TCA cycle intermediates, such as fumarate, succinate, and malate, among the groups. This could imply that reduced mitochondrial activity may lead to a decrease in ATP production [134]. Furthermore, the authors propose that reduced levels of ATP in long COVID patients could potentially hinder the tissue repair process, leading to prolonged inflammation. Thus, enhancing ATP levels through the use of ATP regulators could be a viable approach to protect cells and tissues in long COVID, as observed in various neurological disorders [144]. Moreover, these patients showed reduced levels of sarcosine and serine, which were inversely correlated with cognitive dysfunction, depression and anxiety. These findings suggest that supplementing with sarcosine and serine could have therapeutic benefits in the management of symptoms in individuals with long COVID [134].

### 4.2. Immune System Impacts of SARS-CoV-2 on Lipid Metabolism

Viruses use their host’s lipid metabolism for replication and immune system evasion while viral-mediated dysregulation of lipid metabolism results in heightened severity of infection [145]. Replication in perinuclear DMVs and trafficking through the ER and Golgi apparatus requires “hijacking” the host’s intracellular lipid metabolism [146]. Post-replicative coronaviruses utilise the host’s endoplasmic reticulum–Golgi intermediate compartment (ERGIC) where new viruses are assembled before they undergo exocytosis from the trans-Golgi network (TGN) [147] allowing viral replication to bypass intracellular immune surveillance [146]. Similarly, SARS-CoV-2 exploits the presence of sphingomyelin-derived ceramide lipid rafts in the cell membrane, which cluster and organise a number of membrane-based receptor molecules including the ACE2 viral port of entry [148,149]. A 2022 study by Kornhuber et al. indicated that SARS-CoV-2 activates acid sphingomyelinase (ASM) inducing increases in ceramide and facilitating viral entry [148].

Patients with low levels of circulating high-density lipoprotein (HDL-C) had lower lymphocyte counts, higher C-reactive protein (CRP) and other acute phase proteins [150]. Two studies have determined that patients with low serum HDL-C suffer a more severe course of COVID-19 infection [150,151]. High serum HDL correlates with good cardiovascular health [152], and a large-scale meta-analysis of over a million subjects concluded that increased HDL is associated with reduced mortality [153]. Clearly, patients with a greater level of overall health prior to infection are more capable of sustained immune response and should have better outcomes, but studies also indicate that acute infection and inflammation cause changes in lipid profile. Decreased HDL-C and increased low-density lipoprotein (LDL-C) and very-low-density lipoprotein (VLDL-C) are seen in patients with acute infection [154], and HDL-C, LDL-C and apolipoprotein-A1 (Apo-A1) have been used as prognostic markers in a variety of disease states [155]. High-density lipoprotein appears to confer protection during inflammation and sepsis and acts as a component of the innate immune system [156]. Reduced formation of Apo-A1 by the liver in inflammation reduces ester formation owing to decreased lecithin-cholesterol acyltransferase (LCAT) activity and increases clearance owing to overproduction of serum amyloid A (SAA), which displaces Apo-A1 in HDL-C and is hypothesised as causing the observed reduction [155]. Increased SAA is associated with increased cardiovascular mortality and appears to be incorporated into HDL-C, rendering the protective effects dysfunctional [157].

Multiple studies now support the hypothesis that acute infection depletes serum cholesterol, activating sterol regulatory element binding protein-2 (SREBP2) [158,159]. This regulatory protein controls lipid cholesterol and fatty acid gene expressions via the MAPK signalling pathway [160]. As the amount of cholesterol within the cell decreases, SREBP2 is increasingly activated, resulting in increased cholesterol production [161]. Indeed, the presence of the C terminal end of SREBP in blood correlates with the severity of disease progression and can be used as an indicator of acute disease prognosis [158]. It is further hypothesised that the reduction in cellular and serum cholesterol acts as an innate immune mechanism preventing viral access to cells—dramatic depletion of cholesterol results in less bond formation between viral S proteins and cellular ACE2 receptors and decreased expression of ACE2 receptors overall [162].

Metabolomic analysis of plasma from PASC patients revealed significantly elevated levels of various types of free and carnitine-conjugated fatty acids; the list comprises saturated, monounsaturated, polyunsaturated, and highly unsaturated fatty acids. These levels were found to be higher compared to those of healthy controls and individuals who had recovered from COVID-19 [34]. Plasma samples from PASC patients exhibited reduced levels of mono-, di-, and tricarboxylates (such as pyruvate, lactate, citrate, succinate, malate) in comparison to the other groups, indicating compromised mitochondrial function and defective fatty acid oxidation [34].

### 4.3. Oxidative Stress

Oxidative stress occurs when the rate of ROS production exceeds the capacity of the anti-oxidant defence system [163] and signs of cellular metabolic and oxidative stress are evident both in acute and post-acute SARS-CoV-2 infection [164]. It plays a role in causing chronic inflammation, dysfunction of the immune system, and damage to mitochondria and endothelial cells [165,166]. SARS-CoV-2 both directly and indirectly impairs mitochondrial function, resulting in increased production of ROS and oxidative stress [167], and involvement of the ACE2 receptor, NADPH oxidases and inflammatory pathways are potential factors in the induction of oxidative stress by SARS-CoV-2 [168]. A reduction in ACE2 receptor expression in infected cells causes upregulation of angiotensin II (ATII). When bound to ACE1 receptors, ATII directly upregulates NADPH oxidase (isoform 2) causing increased ROS production throughout the organism. In physiological conditions, excessive ROS activates Bcl-2-family pro-apoptotic proteins, increasing mitochondrial permeability and releasing caspases, mtDNA and cytochrome c, which can result in programmed death of the cell. Infection by SARS-CoV-2 also interferes with anti-oxidant pathways such as nuclear factor (erythroid-derived 2)-like 2 (NRF2) transcription factor, which controls expression of genes that protect against cellular stress, indirectly resulting in increased intra- and extracellular oxidative stress and overall systemic inflammation [167].

Increased production of ketone bodies within hepatocytes and a failure of oxidative phosphorylation of acetyl-CoA suggests increased metabolic stress throughout the acute phase of SARS-CoV-2 infection and is characterised by increased production of ROS, as are reduced serum levels of essential amino acids, tyrosine and glutamine [154]. Reduced ability to oxidise acetyl-CoA in hepatic mitochondria may also result in significantly increased accumulation of VLDL-C and TAG in serum and indicate further metabolic stress [145]. Similarly, increased expression of the phosphorylated form of H2A histone family member X (γ-H2AX), an intracellular marker of ROS damage to DNA, is seen in tissues expressing viral S protein [169].

A small study of 50 patients in North-West Nigeria found that patients with severe acute infection have a relative deficiency in antioxidant trace elements and vitamins and reduced glutathione and enzymatic antioxidant levels while expressing increased markers of oxidative stress [170]. Patients with increased oxidative stress from prior comorbidities also have a higher rate of severe complication and death upon infection [164], and in an early study in Italy of patients with chronic obstructive pulmonary disease, acute respiratory distress syndrome and severe SARS-CoV-2 infection, all patients were hypoxaemic and displayed an acute imbalance in blood redox state, indicating excess production of ROS [171].

Little-noted but of major interest to the study of PASC is micro-circulatory dysfunction resulting from viral invasion of endothelial tissue contributing to widespread thrombosis in severe disease states [12]. Expression of the S protein in endothelial cell lines caused increased generation of ROS and increased expression of senescence markers indicating metabolic dysfunction, macromolecular damage and cell-cycle arrest [169]. Nevertheless, it remains to be seen whether endothelial dysfunction is caused by the direct invasion of cells or by the action of ROS, cytokines and unbalanced immune response to severe infection [172,173]. What has been determined is that hypoxia in endothelial cells results in increased production of ROS, HIF-1α stabilisation, activation of the PI3K signalling pathway with “Warburg-like” reconfiguration of cellular glucose metabolism, extensive thromboxane activation and micro-circulatory thrombosis [140].

During acute SARS-CoV-2 infection, ROS-mediated damage to the endothelial system results in thrombosis and hyperinflammation, which in turn causes damage to respiratory, cardiovascular, and neurological systems. Increased oxidative stress results in disruption of the immune system, specifically in neutrophil extracellular trap (NET) formation. Neutrophils produce NETs (consisting of DNA and globular proteins) in response to inflammatory cytokines and their function is to provide a matrix for anti-microbial activity. The balance between NET formation and degradation is essential in the prosecution of disease. Excessive NET production results in immuno-thrombotic states, increased susceptibility to sepsis, acute respiratory distress syndrome (ARDS) and acute lung injury. Owing to the high glycaemic burden and the presence of advanced glycation end products, diabetic patients already maintain increased extracellular oxidative stress. Their concurrent, heightened, systemic inflammation explains the more severe course of the disease within this cohort, and similar effects are noted in other groups of patients with inflammatory conditions such as obesity, atherosclerosis and cancer [174]. Meanwhile, the total effect of increased ROS on the immune system remains elusive even though the effects of increased ROS can be seen in certain aspects of immune function. Studies demonstrate the key role of surfactant protein-A (SP-A) on macrophage activation [141,164] and increased presence of ROS causes oxidation of SP-A and a consequent decrease in classical activation of macrophages, resulting in reduced classical immune response and phagocytic activity [175]. Excessive oxygen exposure over a prolonged period is a recognised factor that could increase oxidative stress and potentially play a part in the persistent inflammation that leads to pulmonary fibrosis development post-COVID-19 infection [176]. Numerous studies have demonstrated a link between PASC patients, elevated oxidative toxicity and reduced antioxidant defence. These factors are closely associated with the severity of symptoms such as fatigue, depression, and anxiety [177]. Antioxidant supplements, including high doses of vitamin C administered intravenously, have demonstrated promise in decreasing oxidative stress and improving the recovery of individuals with COVID-19 [174]. More research is necessary to fully comprehend the impact of oxidative stress on PASC and develop targeted therapies.

## 5. Mitochondrial Interference and mtDNA

### 5.1. Computational Modelling

Pioneering work in computational biology conducted with the RNA-GPS machine learning tool in 2020 predicted that SARS-CoV-2 genetic material had a high affinity for residence within host cell mitochondria [178]. Similarly, in 2020 Gordon et al. cloned, tagged and expressed 26 of the 29 SARS-CoV-2 proteins and used affinity purification mass spectrometry (AP-MS) to predict high-confidence interactions between viral and host proteins, determining that viral NSP5 had a high likelihood of interacting with mitochondrial tRNA [179]. A 2014 study on SARS-CoV-1-host interaction pointed to the high possibility of host mitochondrial involvement in viral attempts to evade the innate immune system [180] and it is apparent that SARS-CoV-2 has continued this pattern of behaviour.

SARS-CoV-2 mitochondrial interaction was further demonstrated by Medini et al. in 2021, finding that mitochondrial genes were drastically downregulated in circulating blood cells and immune cells during acute infection but not in respiratory tract tissue [181]. This study highlights the crucial role of mitochondrial-nuclear co-regulation in the COVID-19 immune response. The decrease in mtDNA gene expression in patients’ blood cells indicates a shift towards glycolysis, promoting virus replication [181].

Miller et al. reported after computational analysis of RNA transcriptome data from cell lines, BAL and clinical lung samples that while expression of mitochondrial genes, especially those responsible for proteins of complex I of the electron transport chain and other TCA cycle enzymes, was downregulated, there was no significant effect on MAVS related innate antiviral defence [182]. The evidence indicates that the reduced expression of key mitochondrial genes might be the reason behind energy production difficulties and tissue damage seen in COVID-19 patients.

In contrast to this, during their 2022 study, Li et al. found that expression of viral accessory protein ORF10 caused inhibition of the MAVS-RIG1 pathway and inactivation of IFN type I production [183]. Confusion exists within the literature regarding this topic and more work is required to resolve the question of whether MAVS is inactivated in live, in vivo infection.

### 5.2. Breakthroughs with Fluorescence Microscopy and Multi-Omics

Remarkably, a 2022 study conducted by Shang et al. used fluorescence microscopy to localise SARS-CoV dsRNA (a genetic product of viral replication) within mitochondria, providing a seminal moment in understanding the complex interplay between autophagy, SARS-CoV-2 mitochondrial involvement and the efforts of the virus to evade the innate immune response. The authors noted the association of DMV sites of replication alongside damaged mitochondria and the role of outer membrane transport protein translocase of outer membrane 20 (TOM20) in internalising SARS-CoV-2 dsRNA genetic material, along with the failure of PINK1-Parkin pathway activation to result in successful mitophagy [78]. TOM20 is a component of the translocase of the outer membrane (TOM) complex, responsible for the recognition, import and segregation of most of the precursor proteins required within the organelle [184]. Confirming this, Mozzi et al. determined that the viral accessory protein ORF3c localises itself within the mitochondrion increasing production of mitochondrial ROS yet blocking mitophagy [185]. These studies indicate that SARS-CoV-2 appears capable of sequestration of its genetic material within mitochondria, ensuring that aberrant organelles evade destruction by mitophagy, and this is one hypothesis to explain the greatly increased persistence of viral genetic material and proteins within post-infected patients.

In a wide-ranging 2023 study, Guarnieri et al. used nasopharyngeal biopsies, tissue from autopsies and infected rodent tissue to investigate the effects of SARS-CoV-2 infection on mitochondrial gene transcription [186]. They confirmed RNA-GPS predictions by Wu et al. [178] and Stukalov et al. [187], predicting viral polypeptide binding and interaction with mitochondrial proteins, especially those of ETC complex I, III and IV. Even when the virus was cleared, cardiac, renal, hepatic and lymph node mitochondria remained injured. Mitochondrial bioenergetic gene function was depressed in autopsy samples from the heart, kidneys, liver, lungs and lymph nodes. The degree of mitochondrial gene modulation was proportional to viral load, while post-infection lung samples showed a return to normal expression of oxidative phosphorylation gene expression. Key mitochondrial inner membrane transport enzymes were downregulated in the heart, including SLC25AC phosphate carrier, SLC25A4 and SLC25A6 adenine nucleotide translocases, SLC25A1 citrate carrier and SLC25A12 Ca^2+^ binding aspartate-glutamate carrier, all of which are critical for the correct function of the TCA cycle and oxidative phosphorylation [186]. The ongoing mitochondrial dysfunction observed in various organ systems might account for a number of the persistent symptoms and complications seen in PASC. This is especially relevant for those that pertain to energy metabolism and organ function.

### 5.3. mtDNA as a Prognostic Marker of Acute COVID Infection?

Increased circulating plasma levels of mtDNA have been demonstrated to determine poor outcomes in disease states. Quantitatively analysing systemic circulating mtDNA, Schneck et al. determined that patients suffering from septic shock showed consistently elevated levels when compared with patients suffering from post-operative inflammation [188]. Scozzi et al. demonstrated that high circulating levels of mtDNA were an early indicator of poor outcomes in COVID-19 [107]. Edinger et al. conducted a study of 29 critically ill patients aged between 59 and 80 and found that significantly increased levels of peak plasma mtDNA could be used as a predictive marker of mortality [189]. While the full mechanism remains unclear, circulating mtDNA induces the expression of inflammatory mediators TNF-α and IL-1β in mouse models [190]. Valdés-Aguayo et al. determined that damage to the mitochondrial membrane system during severe acute infection resulted in the release of mtDNA into circulation, triggering innate immune responses. They further concluded that high levels of mtDNA in circulation were useful as markers of infection [191].

According to recent studies, there is evidence to suggest that variations in mtDNA can play a role in determining the risk and severity of SARS-CoV-2 infection (for more information on haplogroups, see the mitomap database at www.mitomap.org, accessed on 11 June 2024). Studies in Slovak COVID-19 patients have shown that specific mtDNA haplogroups were associated with decreased risk of severe COVID-19 (J1, and clusters H + U5b, and T2b + U5b) while other haplogroups (T1, H11 and K (K1a)) were associated with an increased risk of severe COVID-19 [192]. This study recognizes that the limited number of samples and regional differences in mtDNA studies can create biases and impact the findings. The variations in haplogroups within the Han Chinese population of Hubei, China, can be attributed to the presence of different mtDNA variants. Some of these variants, such as C5178a (in NADH dehydrogenase subunit 2), A249d (in the D-loop), T6392C (in the cytochrome C oxidase I gene) and G10310A, have been linked to a decreased risk of severe infection outcomes. On the other hand, variants like A4833G and T3394C have been associated with an increased risk [193]. Based on the results, it can be inferred that specific mtDNA variants have a substantial influence on the functioning of certain OXPHOS enzymes. It is suggested that these mtDNA variations may play a role in determining the severity of COVID-19 and the response to treatments targeting mitochondrial functions or OXPHOS.

Mitochondrial microRNAs (mitomiRs) have vital functions in regulating different aspects of mitochondrial homeostasis, such as controlling fission and fusion, managing mitophagy, regulating mitochondrial calcium levels, influencing OXPHOS, and safeguarding mtDNA integrity [194]. Furthermore, mitomiRs also play a role in regulating mtDNA translation by affecting the translational activity of mitochondrial genomes. A 2019 study of mitomiR-2392 showed that this non-coding RNA regulated chemoresistance in squamous cell carcinomas of the tongue by reprogramming cellular metabolism and downregulating electron transport chain complexes I, III and IV [195]. This fast-evolving field suggests that non-coding and micro-RNA (ncRNA and miRNA) interact at numerous levels to provide immediate control of cellular metabolism and protein function and further study can elucidate their interaction in virally mediated mitochondrial dysfunction [196]. Assessing the functionality of immune cells in the blood through mtDNA and mitomiR monitoring could serve as a non-intrusive approach to diagnose and predict outcomes of SARS-CoV-2 and infection by other viruses.

### 5.4. Mitochondrial Dysfunction in COVID-19 Patients and Therapeutic Implications

The replication of SARS-CoV-2 heavily depends on mitochondria, and the virus–cell interactions lead to disturbances in mitochondrial homeostasis. The investigation of potential treatments for mitochondrial dysfunction in COVID-19 is an active area of research.

The study by Guarnieri et al. highlights the suppression of transcription of certain nuclear DNA-encoded mitochondrial oxidative phosphorylation genes in nasopharyngeal samples, inducing glycolysis and activating immune defence [186]. The decrease in expression of some genes, including glyceraldehyde-3-phosphate dehydrogenase (*GAPDH*) and phosphoglycerate kinase 1 (*PGK1*), in human nasopharyngeal samples can result in the accumulation of substrates in the initial steps of glycolysis. This accumulation promotes increased production of NADPH and fatty acids, and consequently supports viral biogenesis. In contrast, an increase in the expression of the *HIF-1α* gene, glycolysis genes, and *mTOR* signalling genes was observed in the autopsy tissue of patients with COVID-19. Mitochondrial gene expression was recovered in the lungs, yet mitochondrial function remained suppressed in the heart, kidney, and liver. The fact that organs outside the pulmonary system continue to malfunction implies that mitochondrial dysfunction might have a long-term impact on the internal organs of these patients. A significant finding of this study is that impaired mitochondrial gene expression persists in specific tissues even after the virus is cleared, potentially playing a role in the severity of COVID-19 pathology [186]. These findings suggest that therapies aimed at improving mitochondrial function, decreasing excessive mitochondrial reactive oxygen species (mROS), and preventing the release of mitochondrial DNA could potentially be effective in reducing the severity of acute infection by SARS-CoV-2 and relieving symptoms associated with long COVID.

A noteworthy finding in the research conducted by Guarnieri et al. was the detection of a potential therapeutic focus in microRNA 2392, also known as miR-2392. The study revealed that this particular microRNA controls the functioning of mitochondria in the human tissue samples examined [186]. Blocking miR-2392 is suggested as a way to improve mitochondrial function, decrease viral replication, and alleviate the severity of COVID-19 symptoms (Table 1) [197]. microRNA can be also involved in the regulation of inflammatory responses to viral infections [198]. miR-146a and miR-21 are commonly referred to as “inflammamiRs” because they have the capability to control NF-κB-driven inflammatory pathways. They are upregulated in acute and chronic viral infections and specifically target molecules involved in the NF-κB/NLRP3 inflammatory pathways [199]. Modulating their expression could potentially help control inflammation and protect mitochondrial function in COVID-19 patients.

Enhancing our comprehension of the function of mitochondria in SARS-CoV-2 infection could enhance intervention treatments and provide better protection for patients against pathogens. Many potential therapeutic drugs that reduce the severity of human infections with SARS-CoV-2 can be found in the Reactome database (https://reactome.org/content/detail/R-HSA-9679191.1, accessed on 11 June 2024) and the therapeutic implications for mitochondrial dysfunction are presented in Table 1.

## 6. Conclusions

Recent studies suggest clear and significant links between SARS-CoV-2 infection and mitochondrial dysfunction, autophagy, innate immune response and cellular metabolic function. Circulating mtDNA clearly plays a role in acute COVID-19 pathogenesis, yet its role in the pathogenesis of post-acute sequelae and “long COVID” syndrome nevertheless remains elusive. The fact that the PASC-mtDNA association is still not fully understood highlights the need to continue to identify current gaps in our knowledge.

Complex computational multi-omics studies allow metabolic and proteomic profiling of infected tissues, providing a deep understanding of the changes wrought on cellular metabolism by viral infection. In turn, this provides greater access to underlying genetic processes. Understanding the interplay of related processes underlying SARS-CoV-2 replication and pathogenesis allows investigation of the virally induced mitochondrial dysfunction that determines the clinical events of disease progression. New evidence of SARS-CoV-2 RNA sequestration within the host mitochondrial matrix in addition to the nucleolus, predicted by computational models and confirmed in laboratory settings, provides tantalising hints to possible links between mitochondrial and innate immune system dysfunction, viral persistence and post-acute sequelae of COVID-19.

## Figures and Tables

**Table 1:** Therapeutic implications for mitochondrial dysfunction in COVID-19 patients including clinical trials related to assessment and improvement of mitochondrial function in patients with COVID-19 and PASC (from ClinicalTrials.gov, National Library of Medicine, Bethesda, MD, USA).

Target/Strategy		Effect/Hypothesis	Intervention/Treatment	ClinicalTrials.gov Identifier (NCT Number)	Ref.
Antioxidants	N-acetylcysteine (NAC)	By supplementing with glycine and cysteine amino acids (in the form of N-acetylcysteine), it is possible to enhance GSH levels and improve mitochondrial function. As a result, this approach may help to lower oxidative stress, inflammation, and endothelial dysfunction.	Glycine N-acetylcysteine Alanine (placebo)	**NCT04703036**	
	Quercetin Bromelain Zinc Vitamin C	Zinc ionophore may act as antiviral agent, Bromelain is an anti-inflammatory agent, Vitamin C and Quercetin are antioxidants that also stimulate mitochondrial biogenesis	Quercetin Bromelain Zinc Vitamin C	**NCT04468139**	
	Ubiquinol	Unique spa rehabilitation programmes in the High Tatras mountains can help patients with post-COVID-19 syndrome to restore impaired mitochondrial metabolism. The study has the potential to enhance physical and mental activity, boost immunity, reduce oxidative stress, and expedite the recovery process.	Ubiquinol (reduced coenzyme Q10) Mountain spa rehabilitation	**NCT05178225**	
	Mitochondrial-targeted ubiquinone (MitoQ)	MitoQ may help treat COVID-19 by reducing cytokine storms and restoring T cell function through improving mitochondrial dysfunction, which is linked to severe COVID-19 cases. Using MitoQ in the early stages could effectively slow down or postpone the progression of the disease in elderly COVID-19 patients or those with other comorbidities.	MitoQ	-	[200]
	Mitoquinone	The overall objective of this study is to determine whether the daily administration of mito-MES at a dosage of 20 mg is effective in preventing confirmed SARS-CoV-2 infection. The study aims to compare the treatment to a placebo over a 14-day period and will focus on high-risk individuals who have had close contact with confirmed COVID-19 cases.	Mitoquinone/mitoquinol mesylate (mito-MES) Placebo	**NCT05886816**	
	Vitamin C Vitamin E Melatonin N-acetyl cysteine Pentoxifylline	Inclusion of antioxidants like N-acetylcysteine (NAC), vitamin C, melatonin, and vitamin E in the treatment helps enhance intracellular GSH levels, sequester ROS, safeguard cell membrane lipids, cytosol proteins, nuclear DNA, and mitochondria.	Vitamin C Vitamin E Melatonin N-acetyl cysteine Pentoxifylline	**NCT04570254**	
	α-Lipoic acid	ALA has both antioxidant properties and the ability to suppress the NF-kB transcription factor, resulting in the inhibition of cytokine and pro-inflammatory factor production.	NAC (N-acetyl cysteine) α-lipoic acid (ALA) Liposomal glutathione (GSH)	**NCT05371288**	
Mitochondria-targeted therapies	Biogenesis enhancers	Both adults and children with severe COVID-19 and multisystem inflammatory syndrome in children (MIS-C) have been found to have a significant lack of arginine. The limited availability of arginine in the plasma has been suggested as a factor contributing to problems with endothelial function, immune regulation, and excessive blood clotting.	Arginine Hydrochloride	**NCT05855330**	
		The hypothesis proposes that supplementing with L-citrulline (CIT) is superior to ARG administration in correcting hypoargininemia, alleviating lymphocyte dysfunction, rectifying immunosuppression, and improving organ function in septic patients admitted to intensive care.	L-citrulline Placebo (water)	**NCT04404426**	
		Assessment of the effect of drug AXA1135 on improving bioenergetic function (measured via phosphocreatine recovery rate) in patients with fatigue-predominant PASC	AXA1125	**NCT05152849**	[201]
	Mitochondria-protective agents	The study assesses the effect of methylene blue as a broad-spectrum antiviral agent and its stabilising impact on mitochondria.	Methylene blue	-	[202]
	Autophagy modulation	Blocking autophagy in the early phase of COVID-19 infection could potentially control the body’s antiviral IFN response and suppress viral reproduction.	Lysosomotropic agents (e.g., chloroquine, hydroxychloroquine, azithromycin, artemisinin, and imatinib), Protease inhibitors/activating agents (camostat mesylate, lopinavir, ritonavir, umifenovir and teicoplanin), PI3K/AKT/mTOR modulators (e.g., rapamycin, wortmannin)		[203]
	Modulation of the mitochondrial function	The study builds on previous research suggesting that exogenous ketone supplementation can increase mitochondrial respiration in various tissues, including skeletal muscle and adipose tissue	Agilent Seahorse XF Cell Mito Stress Test Ketoneaid (Ketone monoester)	**NCT05798260**	
MicroRNA targeting	miR-2392	SBCov207 aims to mitigate the negative impacts of miR-2392 upregulation observed in COVID-19 patients, including mitochondrial dysfunction, heightened inflammation, increased glycolysis, and hypoxia, by inhibiting miR-2392.	SBCov207- antisense-based therapeutic against human miR-2392	-	[197]
Enhancing the MAVS pathway	Overexpression of MAVS	The use of mesenchymal stem cells to deliver targeted mitochondrial therapy, specifically with an over-expressed MAVS protein, is being explored as a promising new treatment approach for COVID-19. The aim of this approach is to selectively enhance interferon (IFN) production and innate immune responses against SARS-CoV-2.	Mesenchymal stem cells	-	[204]
Immune boosting action	Upregulation of TLR 3	The presence of 13-cis retinoic acid led to a time-dependent upregulation of TLR3, MAVS, and IFN regulatory factor 1. Isotretinoin as “the Immunity passport”	Isotretinoin (13 cis retinoic acid)	**NCT04353180**	
Drug repurposing	Metformin	By modulating mitochondrial function and reducing oxidative stress, metformin may offer potential relief for the cytokine storm and hyperinflammatory response observed in severe cases of COVID-19. Metformin can enhance the function of immune cells by optimizing their energy metabolism.	Metformin Placebo Fluvoxamine Ivermectin	**NCT04510194**	[205]
